# Supplementary material for: Out-of-pocket expenditure on childhood infections and its financial burden on Indian households: Evidence from nationally representative household survey (2017–18)
Source: PLoS One. 2022 Dec 27;17(12):e0278025. doi: 10.1371/journal.pone.0278025 (PMC9794050; doi:10.1371/journal.pone.0278025)

SUPPLEMENTARY MATERIAL

S1 Table. Working definitions of various infectious diseases according to NSSO.

| Sr. No | Disease | Working/Operational Definition |
| --- | --- | --- |
| 1 | Acute Diarrheal Disease/Dysentery/Cholera | Diarrhoea: Passage of 3 or more loose watery stools in the past 24 hours. (with or without vomiting)  Dysentery: Acute diarrhoea with visible blood in the stool.  Cholera: Passage of stools like Rice water, Cold feet, low blood pressure |
| 2 | Viral Hepatitis/Jaundice | Acute illness typically including:  Acute jaundice (Yellowish discoloration of eyes/tongue/nails/palms),  Dark yellow urine,  Reduced food intake,  Severe weakness,  Pain in the right side of upper abdomen |
| 3 | Malaria | A case of fever which may be accompanied with any of the following  Headache,  Backache,  Chills/cold, shivering, sweating,  Muscle pain,  Nausea and vomiting  Convulsions/fits, coma |
| 4 | Dengue Fever | An acute febrile illness of 2-7 days duration with two or more of the following manifestations:  Headache,  Pain behind eyeballs  Muscle pain  Joint pain  Rash,  Bleeding from nose/mouth/under skin |
| 5 | Chikungunya | An acute illness characterised by sudden onset of fever with any of the following symptoms  Headache,  Backache,  Eyes becomes sensitive to light,  Severe pain in joints  Rash |
| 6 | Measles | A person having  Fever ,  Maculopapular rash with cough or running nose or conjunctivitis/redness of eyes |
| 7 | Acute Encephalitis syndrome/Japanese encephalitis | A person of any age, with the acute onset of fever and a change in mental status (symptoms such as confusion, unable to recognize place/person/time, or inability to talk or coma) |

Source: SCH 2017-18

S2 Table: Different types of expenditures incurred by the household for treatment

| Inpatient care | | Outpatient care | |
| --- | --- | --- | --- |
| Medical expenditure | Non-medical expenditure | Medical expenditure | Non-medical expenditure |
| Doctor’s/ surgeon’s fee (hospital staff/ other specialists) | Transport for patient | Doctor’s/ surgeon’s fee (hospital staff/ other specialists) | Transport for patient |
| Medicines | Other non-medical expenses incurred by the household (registration fee, food, transport for others, expenditure on escort, lodging charges if any, etc.) | Medicines: AYUSH | Other expenses incurred by the household (registration fee, food, transport for others, expenditure on escort, etc.) |
| Diagnostic tests |  | Medicines: other than AYUSH |  |
| Bed charges |  | Diagnostic tests |  |
| Other medical expenses (attendant charges, physiotherapy, personal medical appliances, blood, oxygen, etc.) |  | Other medical expenses (attendant charges, physiotherapy, personal medical appliances, blood, oxygen, etc.) |  |

S3 Table: Socio-economic characteristics of children below age 5 years

|  | All children | Children reporting childhoods conditions | Children reporting hospitalisation owing to childhoods conditions |
| --- | --- | --- | --- |
| **Consumption expenditure group** |  |  |  |
| Poorest 20% | 25.69 | 21.36 | 17.12 |
| 2^nd^ poorest 20% | 24.11 | 19.91 | 17.59 |
| Middle 20% | 22.96 | 25.74 | 23.33 |
| 2^nd^ richest 20% | 14.11 | 16.91 | 19.71 |
| Richest 20% | 13.13 | 16.08 | 22.26 |
| **Caste** |  |  |  |
| ST | 10.21 | 9.07 | 6.25 |
| SC | 22.02 | 19.68 | 20.97 |
| OBC | 45.21 | 47.93 | 44.96 |
| Others | 22.56 | 23.32 | 27.83 |
| **Religion** |  |  |  |
| Hindu | 78.38 | 77.38 | 73.96 |
| Muslim | 17.7 | 17.95 | 18.3 |
| Other minority | 3.91 | 4.67 | 7.74 |
| **Main sources of livelihood of households** |  |  |  |
| Self-employed in agriculture | 33.18 | 30.6 | 22.76 |
| Self-employed in non-agriculture | 23.68 | 25.11 | 29.06 |
| Regular wage earning | 12.26 | 14.91 | 15.37 |
| Agriculture labour | 6.99 | 8.12 | 10.22 |
| Casual labour | 20.24 | 18.66 | 19.5 |
| Others | 3.64 | 2.6 | 3.09 |
| **Age (in years)** | |  |  |
| 0 | 19.04 | 17.28 | 19.04 |
| 1 | 10.2 | 14.72 | 19.51 |
| 2 | 19.4 | 21.24 | 21.42 |
| 3 | 20.44 | 18.91 | 14.16 |
| 4 | 20.04 | 16.97 | 16.33 |
| 5 | 10.88 | 10.88 | 9.53 |
| **Gender** |  |  |  |
| Boys | 51.73 | 55.31 | 63.63 |
| Girls | 48.27 | 44.69 | 36.29 |
| N | 70,613 | 4,266 | 3,215 |

Source: Authors estimates using SCH 2017-18

S4 Table: Percentage distribution of all childhood infections by care sought

| Ailment | Formal care | Informal | Self-medicine | No care | Total |
| --- | --- | --- | --- | --- | --- |
| Acute meningitis | 85.81 | 0.84 | 13.34 | 0 | 100 |
| Fever due to diphtheria, whooping cough and tetanus | 78.27 | 1.26 | 20.47 | 0 | 100 |
| Fever with rash/ eruptive lesions* | 79.74 | 6.33 | 13.64 | 0.3 | 100 |
| Tuberculosis | 100 | 0 | 0.00 | 0 | 100 |
| Viral Hepatitis | 92.44 | 6.48 | 1.08 | 0 | 100 |
| Acute diarrhea** | 80.7 | 1.77 | 17.31 | 0.22 | 100 |
| Acute upper respiratory infections*** | 72.08 | 7.09 | 18.45 | 2.38 | 100 |
| Fever with other infections# | 96.56 | 1.83 | 1.61 | 0 | 100 |

Notes: * includes fevers of unknown origin, all specific fevers that do not have a confirmed diagnosis; ** includes dysentery/ increased frequency of stools with or without blood and mucus in stools; *** includes cold, runny nose, sore throat with cough, allergic colds included); # includes malaria and worms infections; $ excluding one outlier case. Source: Authors estimates using SCH 2017-18

S5 Table: Prevalence per 1000 children for childhood infections (age 0-5 years) in outpatient and hospitalization by place of residence

|  | Outpatient care with 15-days recall | | | Hospitalisation with 365-days recall | | |
| --- | --- | --- | --- | --- | --- | --- |
|  | Rural | Urban | Overall | Rural | Urban | Overall |
| Acute meningitis | 6.2 | 6.2 | 6.2 | 1.49 | 2.56 | 1.76 |
| Fever due to diphtheria, whooping cough and tetanus | 5.6 | 7.8 | 6.1 | 0.50 | 1.21 | 0.68 |
| Fever with rash * | 40.5 | 54.2 | 44.0 | 6.67 | 11.46 | 7.89 |
| Tuberculosis | 0.1 | 0.0 | 0.1 | 0.07 | 0.05 | 0.06 |
| Viral Hepatitis | 0.2 | 0.2 | 0.2 | 1.80 | 1.61 | 1.75 |
| Acute diarrhea** | 3.4 | 3.8 | 3.5 | 2.21 | 2.65 | 2.32 |
| Acute upper respiratory infections*** | 15.4 | 21.2 | 16.8 | 1.50 | 1.59 | 1.53 |
| Fever with other infections# | 0.6 | 0.5 | 0.6 | 0.89 | 0.56 | 0.81 |
| All Infectious diseases | 72.1 | 93.9 | 77.6 | 15.13 | 21.69 | 16.80 |

Notes: * includes fevers of unknown origin, all specific fevers that do not have a confirmed diagnosis; ** includes dysentery/ increased frequency of stools with or without blood and mucus in stools; *** includes cold, runny nose, sore throat with cough, allergic colds included); # includes malaria and worms infections. Source: Authors estimates using SCH 2017-18

S6 Table: Distribution of care sought for childhood infections (age 0-5 years) in outpatient settings by place of residence

|  | Outpatient care with 15-day recall | | |
| --- | --- | --- | --- |
|  | Rural | Urban | Overall |
| Acute meningitis | 89.16 | 75.93 | 85.8 |
| Fever due to diphtheria, whooping cough and tetanus | 76.18 | 82.67 | 78.3 |
| Fever with rash * | 75.61 | 88.76 | 79.7 |
| Tuberculosis | 100 | 100 | 100 |
| Viral Hepatitis | 90.27 | 100 | 92.4 |
| Acute diarrhea** | 75.67 | 93.86 | 80.7 |
| Acute upper respiratory infections*** | 67.14 | 82.6 | 72.1 |
| Fever with other infections# | 96.07 | 98.42 | 96.6 |
| All Infectious diseases | 75.29 | 86.3 | 78.68 |

Notes: * includes fevers of unknown origin, all specific fevers that do not have a confirmed diagnosis; ** includes dysentery/ increased frequency of stools with or without blood and mucus in stools; *** includes cold, runny nose, sore throat with cough, allergic colds included); # includes malaria and worms infections. Source: Authors estimates using SCH 2017-18

S7 Table: Per episode OOPE (INR) on outpatient and hospitalization care for childhood infections in India, 2018 by place of residence.

|  | Outpatient care with 15-days recall | | | | | | Hospitalisation with 365-days recall | | | | | |
| --- | --- | --- | --- | --- | --- | --- | --- | --- | --- | --- | --- | --- |
|  | Rural | | | Urban | | | Rural | | | Urban | | |
|  | Medical | Non-medical | Total | Medical | Non-medical | Total | Medical | Non-medical | Total | Medical | Non-medical | Total |
| Acute meningitis | 421 | 106 | 527 | 1037 | 83 | 1120 | 4785 | 788 | 5895 | 6706 | 595 | 7489 |
| Fever due to diphtheria, whooping cough and tetanus | 411 | 95 | 506 | 701 | 48 | 749 | 5901 | 813 | 7050 | 10279 | 1056 | 11610 |
| Fever with rash * | 388 | 82 | 470 | 548 | 67 | 615 | 9444 | 855 | 10628 | 9041 | 936 | 10260 |
| Tuberculosis | 943 | 175 | 1118 | 1800 | 200 | 2000 | 4785 | 931 | 6143 | 869 | 2380 | 3358 |
| Viral Hepatitis | 1194 | 361 | 1555 | 2510 | 372 | 2882 | 12569 | 1029 | 14057 | 14873 | 804 | 15948 |
| Acute diarrhea** | 603 | 71 | 674 | 618 | 80 | 698 | 4951 | 619 | 5887 | 6633 | 647 | 7485 |
| Acute upper respiratory infections*** | 336 | 48 | 384 | 359 | 41 | 399 | 5689 | 1004 | 7089 | 10937 | 1023 | 12157 |
| Fever with other infections# | 589 | 141 | 730 | 1195 | 86 | 1281 | 5451 | 926 | 6693 | 10517 | 881 | 11653 |

Notes: * includes fevers of unknown origin, all specific fevers that do not have a confirmed diagnosis; ** includes dysentery/ increased frequency of stools with or without blood and mucus in stools; *** includes cold, runny nose, sore throat with cough, allergic colds included); # includes malaria and worms infections. Source: Authors estimates using SCH 2017-18

S8 Table: Per episode OOPE (INR) on outpatient care for different components of treatment for childhood infections in India, 2018

|  | Medical expenditure (INR) | | | | | Non-medical expenditure (INR) | | |
| --- | --- | --- | --- | --- | --- | --- | --- | --- |
| Ailment and treatment | Consultancy fee | Medicines | Diagnostic | Other medical | Total medical | Transport | Other non-medical | Total non-medical^##^ |
| **Public** |  |  |  |  |  |  |  |  |
| Acute meningitis | 3.0 | 61.6 | 3.6 | 11.5 | 79.8 | 60.1 | 44.2 | 104.4 |
| Fever due to diphtheria, whooping cough and tetanus | 2.2 | 258.1 | 7.5 | 18.4 | 286.1 | 41.0 | 75.1 | 116.1 |
| Fever with rash* | 5.3 | 105.3 | 18.0 | 4.1 | 132.7 | 39.2 | 43.8 | 83.0 |
| Tuberculosis | 0.0 | 326.4 | 0.0 | 0.0 | 326.4 | 2.7 | 1.4 | 4.1 |
| Viral Hepatitis | 0.0 | 216.9 | 61.6 | 51.0 | 329.6 | 74.4 | 62.8 | 137.2 |
| Acute diarrhea** | 87.2 | 279.7 | 33.2 | 36.3 | 436.4 | 45.7 | 56.8 | 102.5 |
| Acute upper respiratory infections*** | 6.0 | 122.1 | 9.7 | 5.9 | 143.7 | 44.7 | 28.7 | 73.4 |
| Fever with other infections# | 20.1 | 292.0 | 19.6 | 6.7 | 338.4 | 65.2 | 29.0 | 94.2 |
| Total | 9.6 | 132.5 | 15.1 | 8.5 | 165.8 | 43.0 | 44.7 | 87.7 |
| **Private** |  |  |  |  |  |  |  |  |
| Acute meningitis | 138.9 | 592.7 | 47.2 | 24.9 | 803.7 | 47.6 | 58.1 | 105.7 |
| Fever due to diphtheria, whooping cough and tetanus | 146.3 | 410.8 | 75.4 | 10.7 | 643.2 | 44.8 | 30.0 | 74.8 |
| Fever with rash* | 128.8 | 381.4 | 40.9 | 25.3 | 576.4 | 44.5 | 40.4 | 84.9 |
| Tuberculosis | 302.0 | 705.8 | 116.0 | 0.0 | 1123.8 | 97.2 | 103.2 | 200.5 |
| Viral Hepatitis | 290.4 | 1162.1 | 261.9 | 167.8 | 1882.2 | 207.4 | 233.8 | 441.2 |
| Acute diarrhea** | 166.0 | 428.3 | 66.5 | 12.2 | 673.0 | 34.5 | 36.6 | 71.0 |
| Acute upper respiratory infections*** | 95.2 | 321.2 | 28.3 | 3.5 | 448.3 | 26.6 | 20.2 | 46.8 |
| Fever with other infections# | 179.7 | 609.0 | 123.6 | 28.6 | 940.8 | 73.6 | 76.6 | 150.2 |
| Total | 126.4 | 394.6 | 43.6 | 19.6 | 584.1 | 41.4 | 37.7 | 79.1 |
| **Public+Private** |  |  |  |  |  |  |  |  |
| Acute meningitis | 98.0 | 427.7 | 32.1 | 19.6 | 577.3 | 50.3 | 49.9 | 100.2 |
| Fever due to diphtheria, whooping cough and tetanus | 88.1 | 344.0 | 57.0 | 15.0 | 504.0 | 37.2 | 42.7 | 79.9 |
| Fever with rash* | 88.8 | 298.0 | 32.5 | 17.8 | 437.1 | 39.4 | 37.4 | 76.8 |
| Tuberculosis | 266.8 | 661.6 | 102.4 | 0.0 | 1030.9 | 86.2 | 91.4 | 177.6 |
| Viral Hepatitis | 216.2 | 916.6 | 208.6 | 136.5 | 1477.9 | 173.8 | 188.6 | 362.4 |
| Acute diarrhea** | 129.2 | 412.6 | 46.4 | 16.2 | 604.4 | 37.2 | 35.6 | 72.7 |
| Acute upper respiratory infections*** | 64.0 | 253.7 | 19.3 | 6.4 | 343.4 | 25.8 | 19.9 | 45.7 |
| Fever with other infections^#^ | 121.3 | 486.7 | 84.6 | 21.7 | 714.3 | 70.0 | 59.5 | 129.4 |
| Total | 86.7 | 311.1 | 33.1 | 15.5 | 446.5 | 37.7 | 35.6 | 73.3 |

Notes: * includes fevers of unknown origin, all specific fevers that do not have a confirmed diagnosis; ** includes dysentery/ increased frequency of stools with or without blood and mucus in stools; *** includes cold, runny nose, sore throat with cough, allergic colds included); # includes malaria and worms infestations. ##Other non-medical includes food and lodging charge for patient and accompanying person. Source: Authors estimates using SCH 2017-18

S9 Table: Per episode OOPE (INR) on hospitalisation for different components of treatment for childhood infections in India, 2018

| Ailment | Package lump-sum | Consultancy fee | Medicines | Diagnostic | Bed charges | Other medical | Total medical | Transport | Other nonmedical^##^ | Total non-medical |
| --- | --- | --- | --- | --- | --- | --- | --- | --- | --- | --- |
| **Public** |  |  |  |  |  |  |  |  |  |  |
| Acute meningitis | 0.7 | 2.2 | 553.9 | 109.7 | 3.1 | 379.7 | 1049.2 | 268.6 | 620.1 | 888.7 |
| Fever due to diphtheria, whooping cough and tetanus | 1.2 | 14.4 | 1276.8 | 355.6 | 10.9 | 99.7 | 1758.6 | 353.5 | 879.0 | 1232.5 |
| Fever with rash * | 77.1 | 191.3 | 1634.1 | 1583.9 | 586.5 | 517.1 | 4589.8 | 243.2 | 684.5 | 927.7 |
| Tuberculosis | 1058.2 | 2875.9 | 4622.7 | 1861.5 | 862.8 | 1738.4 | 13019.5 | 482.0 | 2020.2 | 2502.2 |
| Viral Hepatitis | 172.3 | 124.7 | 1295.5 | 234.4 | 26.8 | 323.2 | 2177.0 | 284.0 | 656.8 | 940.8 |
| Acute diarrhea** | 1.7 | 3.8 | 956.9 | 140.8 | 3.0 | 145.7 | 1252.0 | 257.4 | 471.2 | 728.6 |
| Acute upper respiratory infections*** | 0.0 | 10.4 | 1079.2 | 268.4 | 20.3 | 146.7 | 1525.0 | 454.0 | 1237.3 | 1691.3 |
| Fever with other infections# | 176.4 | 26.7 | 1129.5 | 397.8 | 13.5 | 164.6 | 1908.4 | 279.4 | 685.7 | 965.1 |
| Total | 67.7 | 117.9 | 1290.4 | 793.1 | 256.8 | 358.1 | 2884.0 | 281.3 | 710.0 | 991.3 |
| **Private** |  |  |  |  |  |  |  |  |  |  |
| Acute meningitis | 1838.3 | 1579.7 | 2325.8 | 768.6 | 1441.0 | 427.7 | 8381.1 | 254.0 | 677.7 | 931.7 |
| Fever due to diphtheria, whooping cough and tetanus | 609.1 | 2116.1 | 3033.5 | 1550.3 | 2727.7 | 1771.0 | 11807.8 | 298.2 | 1105.8 | 1404.0 |
| Fever with rash * | 3342.0 | 1615.1 | 2570.5 | 1071.3 | 2056.8 | 752.0 | 11407.6 | 352.7 | 979.6 | 1332.3 |
| Tuberculosis | 374.5 | 831.9 | 2088.0 | 895.8 | 2540.6 | 460.5 | 7191.3 | 456.0 | 1716.7 | 2172.7 |
| Viral Hepatitis | 7566.0 | 2249.7 | 3282.7 | 1557.1 | 3695.6 | 1490.2 | 19841.4 | 524.1 | 1161.3 | 1685.5 |
| Acute diarrhea** | 1499.4 | 1183.8 | 2421.5 | 926.4 | 1539.1 | 518.3 | 8088.6 | 309.3 | 738.0 | 1047.3 |
| Acute upper respiratory infections*** | 2284.4 | 1338.7 | 2563.2 | 1178.8 | 2294.0 | 656.8 | 10315.9 | 279.9 | 880.1 | 1160.0 |
| Fever with other infections# | 499.2 | 1509.0 | 2701.9 | 1062.8 | 2092.5 | 649.3 | 8514.8 | 314.5 | 1037.7 | 1352.2 |
| Total | 3028.0 | 1606.2 | 2619.2 | 1097.2 | 2139.1 | 786.3 | 11276.0 | 344.0 | 937.4 | 1281.4 |
| **Public+Private** |  |  |  |  |  |  |  |  |  |  |
| Acute meningitis | 1082.8 | 931.1 | 1597.3 | 497.7 | 849.8 | 407.9 | 5366.6 | 260.0 | 654.0 | 914.0 |
| Fever due to diphtheria, whooping cough and tetanus | 354.0 | 1234.0 | 2296.1 | 1048.8 | 1587.4 | 1069.5 | 7589.8 | 321.4 | 1010.6 | 1332.0 |
| Fever with rash * | 2277.3 | 1150.8 | 2265.2 | 1238.4 | 1577.3 | 675.4 | 9184.4 | 317.0 | 883.4 | 1200.4 |
| Tuberculosis | 850.2 | 2254.1 | 3851.6 | 1567.7 | 1373.2 | 1349.6 | 11246.5 | 474.1 | 1927.9 | 2401.9 |
| Viral Hepatitis | 4512.6 | 1372.1 | 2462.0 | 1010.9 | 2180.5 | 1008.3 | 12546.5 | 425.0 | 952.9 | 1377.9 |
| Acute diarrhea** | 860.9 | 680.8 | 1797.1 | 591.5 | 884.3 | 359.5 | 5174.0 | 287.2 | 624.2 | 911.4 |
| Acute upper respiratory infections*** | 1414.3 | 832.8 | 1998.0 | 832.0 | 1428.0 | 462.5 | 6967.6 | 346.2 | 1016.1 | 1362.4 |
| Fever with other infections# | 390.6 | 1010.2 | 2172.8 | 839.0 | 1392.9 | 486.2 | 6291.6 | 302.7 | 919.2 | 1222.0 |
| Total | 1937.0 | 1057.7 | 2129.5 | 985.1 | 1445.3 | 628.5 | 8183.0 | 320.9 | 853.6 | 1174.5 |

Notes: * includes fevers of unknown origin, all specific fevers that do not have a confirmed diagnosis; ** includes dysentery/ increased frequency of stools with or without blood and mucus in stools; *** includes cold, runny nose, sore throat with cough, allergic colds included); # includes malaria and worms infestations. ##Other non-medical includes food and lodging charge for patient and accompanying person. Source: Authors estimates using SCH 2017-18

S10 Table: Average share of household consumption expenditure incurred on treatment of childhood infections (age 0-5 years) in outpatient and hospitalization by different quintiles by insurance

|  | OOPE share (%) on outpatient with 15-days recall | | OOPE share (%) on hospitalization with 365-days recall | |
| --- | --- | --- | --- | --- |
| Quintile groups of households* | Without Insurance | With Insurance | Without Insurance | With Insurance |
| Poorest 20% (Q1) | 8.0 (0.5) | 8.1 (1.7) | 8.7 (0.8) | 5.2 (0.7) |
| 2nd poorest 20% (Q2) | 5.3 (0.4) | 5.8 (0.7) | 11.2 (1.3) | 5.0 (0.6) |
| Middle 20% (Q3) | 4.2(0.2) | 6.2 (0.8) | 5.7 (0.4) | 5.9 (0.8) |
| 2nd richest 20% (Q4) | 4.1 (0.3) | 2.8 (0.2) | 5.3 (0.3) | 11.0 (1.7) |
| Richest 20% (Q5) | 2.8 (0.2) | 2.7 (0.3) | 4.8 (0.3) | 6.2 (1.7) |
| Overall | 4.9 (0.2) | 4.5 (0.3) | 6.8 (0.3) | 6.6 (0.4) |

Note: * based on households’ total consumption expenditure. The numbers in the parenthesis depict standard errors.

Source: Authors estimates using SCH 2017-18

S1 Figure A-I. Outpatient OOPE on childhood infections as a share of monthly per capita monthly consumption expenditure of households.


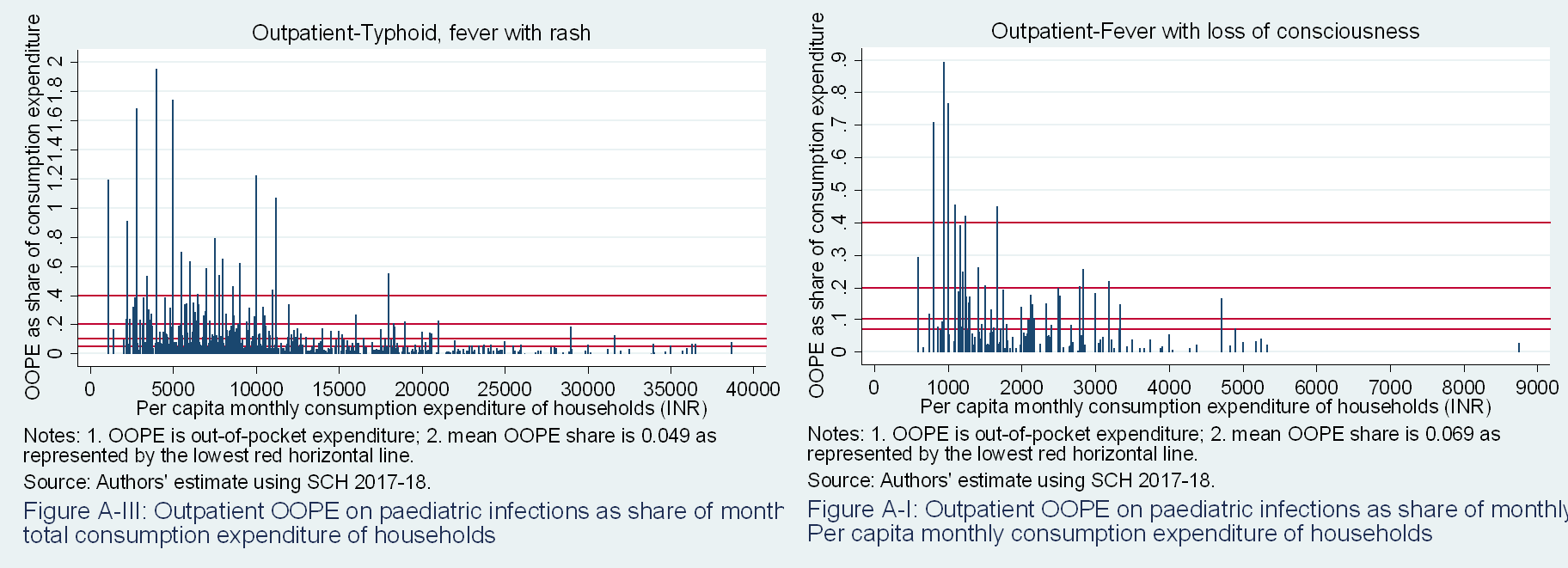


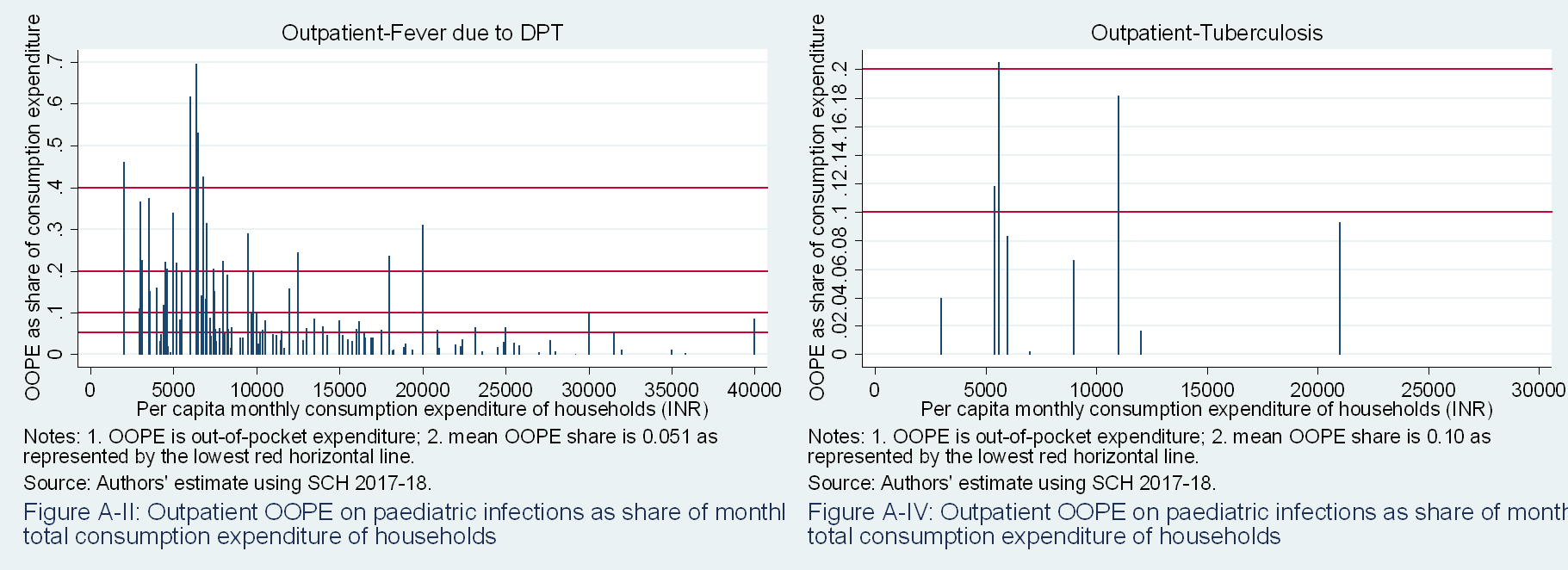


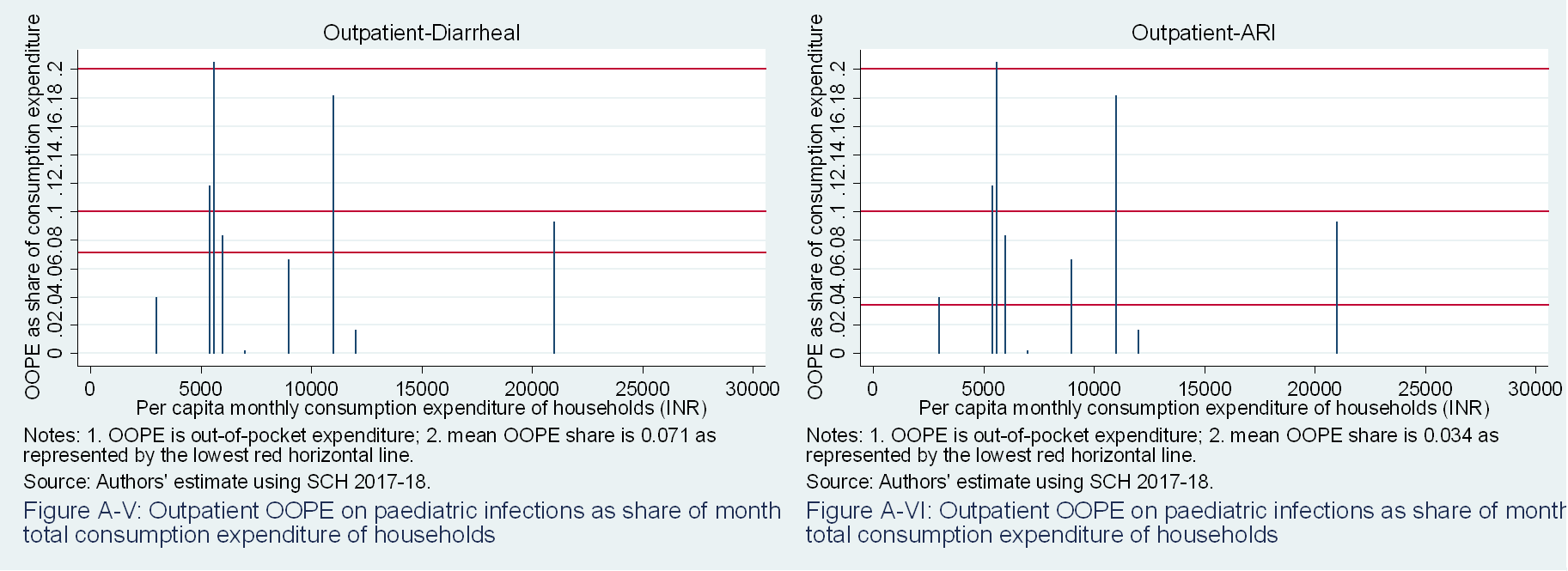


S1 Figure A-II. Inpatient OOPE on childhood infections as share of monthly total consumption expenditure of households.


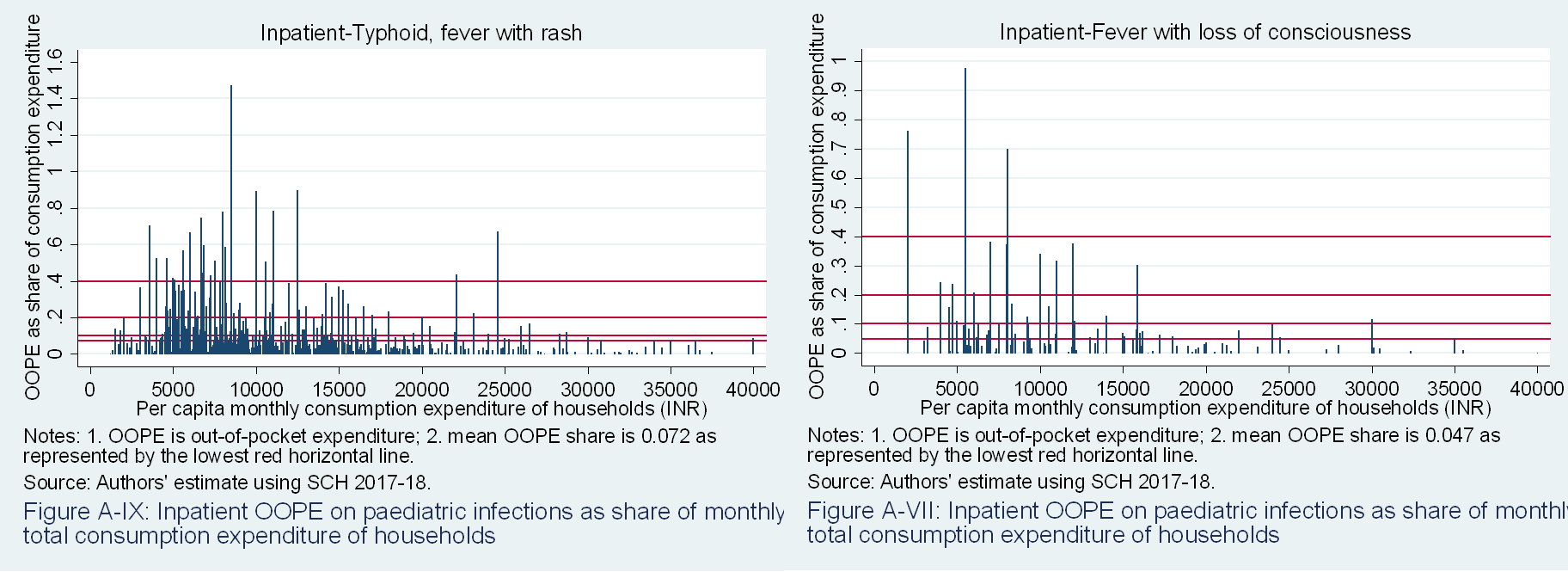


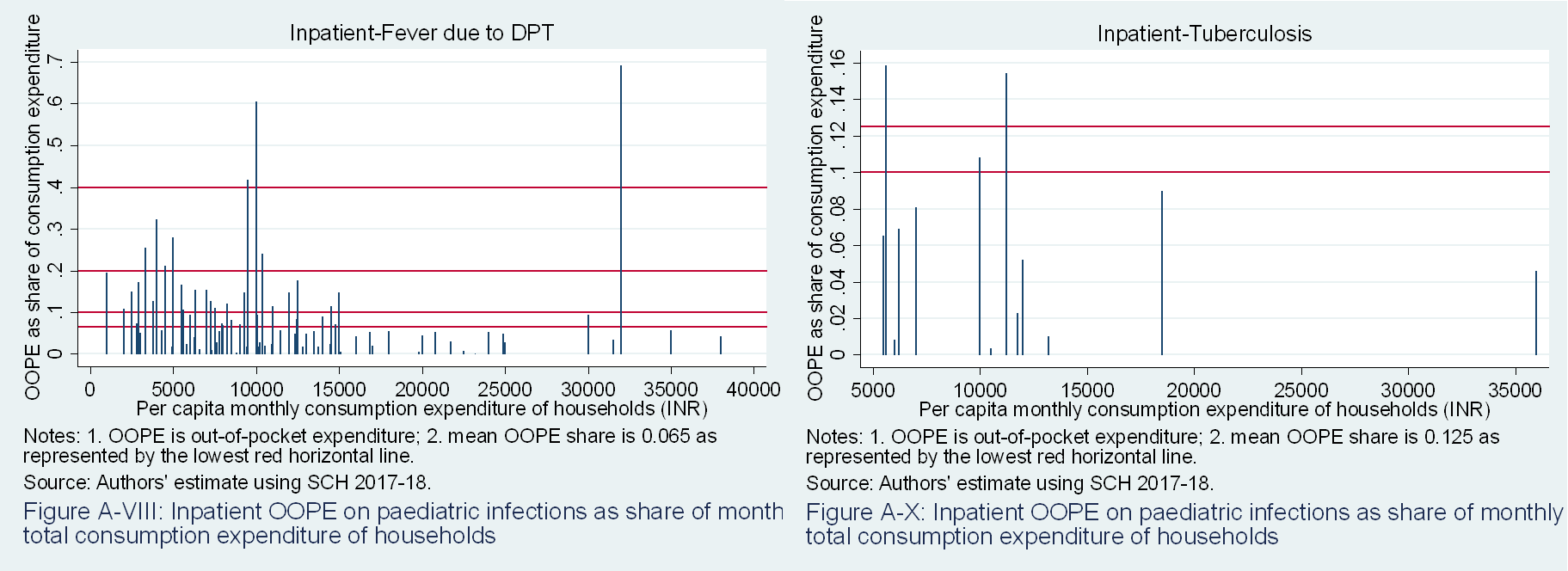


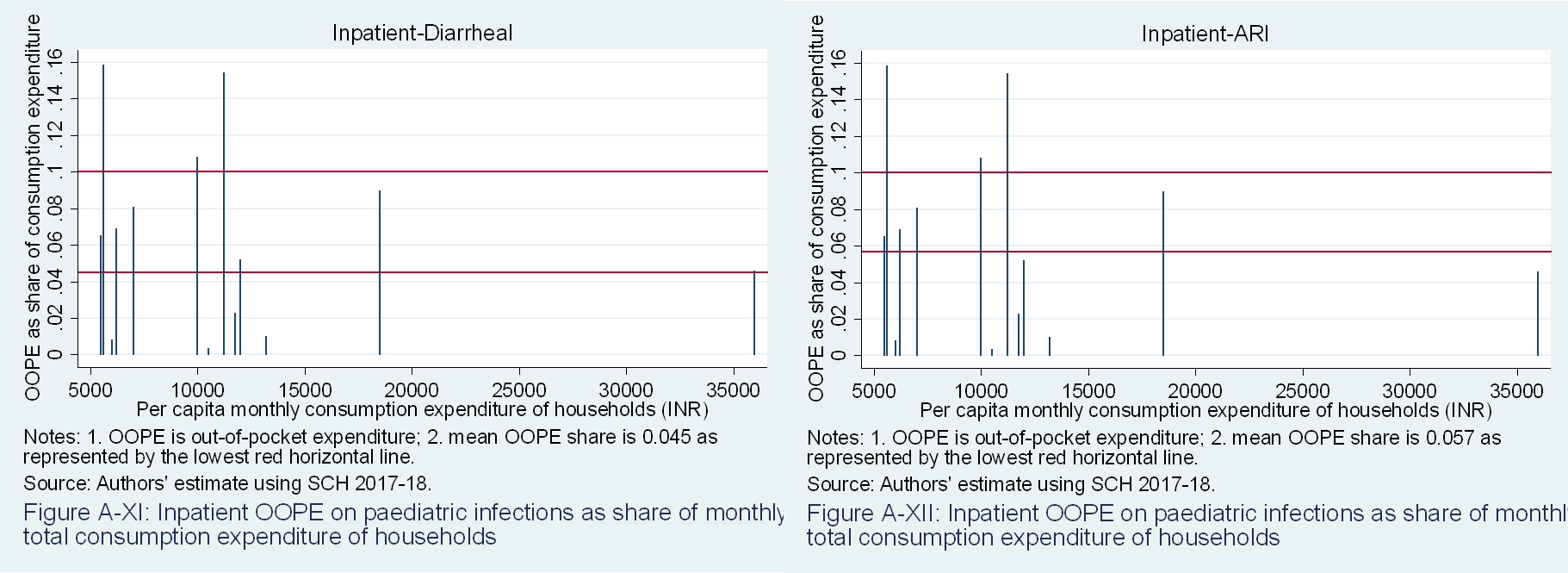

Supplement: S1 File — (DOCX) [file pone.0278025.s001.docx]
