# Supplementary material for: Out-of-pocket expenditure on childhood infections and its financial burden on Indian households: Evidence from nationally representative household survey (2017–18)
Source: PLoS One. 2022 Dec 27;17(12):e0278025. doi: 10.1371/journal.pone.0278025 (PMC9794050; doi:10.1371/journal.pone.0278025)
Supplement: Attachment — Submitted filename: Reviewer Comments.docx [file pone.0278025.s002.docx]

**Reviewer comments**

The present study estimates the direct medical and non-medical expenditure incurred by the households on childhood infections and equity related impact. I congratulate the authors in successfully undertaking this analysis. Although, overall, the study methodology and analysis looks great, I have a few concerns as follows:

1. The analysis reports and highlights higher OOPE (both for outpatient and inpatient care) in private facilities as compared to public hospitals. Though, this is an apparent finding, because in the context of India, whole of the treatment expenses are paid out of pocket for getting treatment in private hospitals (if a patient does not have any sort of pre-payment risk pooling mechanism), whereas in public hospitals, treatment is subsidised by the government and thus only some of the proportion of the total expenditure is paid out of pocket. It would be nice to provide component wise breakdown (Doctor fee, medicine, diagnostics, travel, food, etc.) of the direct medical and non-medical expenditure, separately for public and private hospitals. This would point out that on which specific component, the proportion of OOPE is the highest. This would also support one of the conclusions of the study that “public health system needs strengthening in terms of diagnostics and medicines”.
2. The authors did not estimate the impact of insurance on treatment expenses incurred by the families. Considering, the presence of various state level and centrally sponsored public health scheme (even before the launch of AB-PMJAY) across India during the time of data collection, it will be exciting to see whether those insured households had a lower OOP expenses or better financial risk protection as compared to uninsured ones during the event of hospitalization. Further, it will also be interesting to have some discussion around this issue.

On one side, where public hospitals are being funded through supply side financing mechanisms and also strengthened through demand side mechanisms (publicly sponsored insurance), the households are still incurring OOP expenses. Even in private hospitals, those covered through insurance (especially the poorest quintiles), are also not expected to pay any money from the pocket. Considering that the Government is currently more focussed and investing a huge sum of money in the publicly sponsored insurance schemes, it would be great if authors can comment a bit around the impact of insurance in terms of reduction of OOPE. The authors report that poorer have higher extent of financial risk. It would also be interesting to see the effect of insurance specifically among the poorer sections.

3. The authors had used ‘OOPE as a share of household’s total consumption expenditure’ as a measure of financial risk protection. While calculating financial risk, the standard approach is to express OOPE as a proportion of a household’s capacity-to-pay, which is typically represented by non-food consumption expenditure. Since, the richer households often tend to spend a higher proportion of their total expenditure on health, the measure used in this study can be pro-rich. Though, I am fine with the approach used by authors, I would still recommend using the standard methodology.
4. In table 5, the authors show that the disease related OOPE burden was disproportionately higher for the poorest 20% of households (outpatient, 7.9%; hospitalization, 8.2%) as compared to richer quintiles. I would recommend providing a ‘p value’ and showing whether this difference in OOPE across the quintile groups was statistically significant or not. Similarly, a ‘p-value’ for showing difference in OOP across quintile in each of the disease group in fig 2a and 2b should be provided. Also recommend providing p value for table 4.
5. It would be nice to add SE (standard error) alongside mean OOPE in table 2 and table 3.
6. The figures 1a, 1b and 2b, shows that share of OOP expenses among some of the households were even more than the 100% of their consumption expenditure. The authors could also report on the various coping mechanisms undertaken by these households for dealing with such expenses incurred.
7. To make analysis more comprehensive, it would be nice add a section showing urban-rural differences in term of prevalence, utilization and OOPE due to the childhood infectious diseases.
8. In the section of ‘Statistical analysis’, the authors mention that: “We estimated total OOPE for households by summing up OOPE on all episodes of outpatient visits and hospitalisation, for children aged less than 5 years, and averaged over 30 days”. Was the averaging done for those specific cases that had both outpatient and hospitalization event? **Not very clear**.
9. In the results section in fig 1a and 1b, OOPE as a proportion of consumption expenditure across different levels of thresholds of more than 10%, 20% or 40% of total household consumption expenditure has been reported. It would be more informative to provide an exact estimate of proportion of households crossing the different levels of threshold (i.e., 10%, 20% and 40%). Lastly, it would also make sense, to run a regression analysis (may be logistic regression) to examine the risk of incurring OOPE as a proportion of (e.g.,) more than the 40% of household’s capacity to pay, with covariate including the type of illness, presence of insurance, type of provider, income quintiles, sex, age, etc.
